# A new platelet cryoprecipitate glue promoting bone formation after ectopic mesenchymal stromal cell-loaded biomaterial implantation in nude mice

**DOI:** 10.1186/scrt149

**Published:** 2013-01-04

**Authors:** Marina Trouillas, Marie Prat, Christelle Doucet, Isabelle Ernou, Corinne Laplace-Builhé, Patrick Saint Blancard, Xavier Holy, Jean-Jacques Lataillade

**Affiliations:** 1 Department of Research, ≪Centre de Transfusion Sanguine des Armées Jean Julliard≫, 1 rue Lieutenant Raoul Batany, Clamart, 92141, France; 2 Celogos, 15 rue Beranger, Paris, 75003, France; 3 UMR8081-IRCIV, Institut Gustave Roussy, 114 rue Edouard Vaillant, Villejuif 94805, France; 4 Service Anatomie-Pathologie, Hôpital Percy, 101 avenue Henri Barbusse, Clamart, 92141, France; 5 Département de Physiologie Aérospatiale, Institut de Recherche Biomédicale des Armées, Brétigny sur orge, 91223, France; 6 Ecole du Val de Grâce, 1 Place Alphonse Laveran, Paris, 75005, France

## Abstract

**Introduction:**

This study investigated the promising effect of a new Platelet Glue obtained from Cryoprecipitation of Apheresis Platelet products (PGCAP) used in combination with Mesenchymal Stromal Cells (MSC) loaded on ceramic biomaterials to provide novel strategies enhancing bone repair.

**Methods:**

PGCAP growth factor content was analyzed by ELISA and compared to other platelet and plasma-derived products. MSC loaded on biomaterials (65% hydroxyapatite/35% beta-TCP or 100% beta-TCP) were embedded in PGCAP and grown in presence or not of osteogenic induction medium for 21 days. Biomaterials were then implanted subcutaneously in immunodeficient mice for 28 days. Effect of PGCAP on MSC was evaluated *in vitro *by proliferation and osteoblastic gene expression analysis and *in vivo *by histology and immunohistochemistry.

**Results:**

We showed that PGCAP, compared to other platelet-derived products, allowed concentrating large amount of growth factors and cytokines which promoted MSC and osteoprogenitor proliferation. Next, we found that PGCAP improves the proliferation of MSC and osteogenic-induced MSC. Furthermore, we demonstrated that PGCAP up-regulates the mRNA expression of osteogenic markers (Collagen type I, Osteonectin, Osteopontin and Runx2). *In vivo*, type I collagen expressed in ectopic bone-like tissue was highly enhanced in biomaterials embedded in PGCAP in the absence of osteogenic pre-induction. Better results were obtained with 65% hydroxyapatite/35% beta-TCP biomaterials as compared to 100% beta-TCP.

**Conclusions:**

We have demonstrated that PGCAP is able to enhance *in vitro *MSC proliferation, osteoblastic differentiation and *in vivo *bone formation in the absence of osteogenic pre-induction. This clinically adaptable platelet glue could be of interest for improving bone repair.

## Introduction

Despite years of ongoing research, reconstruction of large bone defects, resulting from a variety of pathological events (trauma and surgical treatment of tumors), remains a challenging clinical problem. Classic therapeutic approaches rely on autologous cancellous bone graft, which today is considered the 'gold standard' treatment [1]. However, the use of autograft, which often results in a favorable clinical outcome, is limited in quantity and is associated with complications at the harvesting site [2]. Bone tissue engineering has emerged as a promising approach based on the elaboration of a bone substitute mimicking bone autograft, which contains all of the key components required for bone repair: (a) an osteoconductive scaffold, (b) cells with osteogenic potential, and (c) growth factors for osteoinduction and vascularization [3].

The main outcomes for a biomaterial are to fill the defect, to replace bone in the short term, and to favor bone formation and remodeling in the long term. The fine tuning of both porosity and chemical composition should be optimal to allow cell migration, proliferation, differentiation, vascular invasion without inducing immune rejection and degradation with time in non-toxic products. Among a large assortment of three-dimensional scaffolds, calcium phosphate ceramics, which possess excellent biocompatibility due to their close resemblance to bone mineral, should be a good candidate for bone tissue engineering [4,5].

Adult mesenchymal stromal cells (MSCs) [6], fibroblast-like cells that can be isolated from bone marrow or other connective tissues [7], can be easily expanded *in vitro*. These cells are further characterized by their multi-potential capacity for differentiation into osteoblasts, chondrocytes, and adipocytes [8]. Thus, the homing ability of MSCs to injury sites, their paracrine secretions enhancing cell migration, differentiation, or angiogenesis as well as their immunomodulatory properties make them an ideal cell type for bone repair [9]. *In vivo*, MSCs coming from a perivascular site or periosteum are involved in direct or secondary fracture healing by their osteogenic and chondrogenic differentiation potential [10]. Locally injected or pre-seeded MSCs on different kinds of biomaterials can enhance the repair of critical size defect in animal models [11,12]. Several teams have obtained significant clinical results after MSC allogenic graft in osteogenesis imperfecta [13,14] or after implantation of hydroxyapatite biomaterials loaded with autologous MSCs [15,16].

The third important fate for bone healing is osteoinduction. Platelets play an essential role in wound healing and are the main regulators of the inflammatory phase. In fact, upon activation, platelets release growth factors and inflammatory molecules from their granule α. These molecules have been shown to enhance MSC proliferation and differentiation *in vitro *[17], angiogenesis, fibroblast proliferation, and extracellular matrix deposition [18,19]. Thus, administration of platelet concentrates named platelet-rich plasma (PRP) has been evaluated in several pre-clinical and clinical studies demonstrating their capacity to improve, for example, dental implant surgery, orthopedic surgery, muscle and tendon repair, and skin ulcers [20-22]. Controversial results in the field of bone repair have been obtained with the use of platelet-derived products [23-26] and could be explained by the diversity of preparations and terms of use. Despite these discrepancies, MSCs combined to platelet-derived products with or without biomaterials seem to improve bone healing [27,28].

Various studies were performed to improve osteoinduction processes such as *in vitro *chemical pre-conditioning [29], *in vitro *platelet lysate (PL) priming [30,31], or *in vitro *osteogenic pre-induction [32] of implanted MSCs. However, the exact mechanisms of action of these *in vitro *preparations on *in vivo *bone formation are not well understood. Furthermore, the interactions between the various components used (biomaterial, cells, and osteoinductive factors) have to be investigated. For this purpose, we used an ectopic model of bone formation in which it is possible to investigate various actors involved in bone formation [33].

In the present study, we first developed a simple innovative process that is clinically adaptable, producing a platelet glue obtained from cryoprecipitation of apheresis platelet products (PGCAP), highly concentrated in fibrinogen and growth factors. Next, we studied whether MSCs pre-cultured with or without osteoblast-inductors loaded on various types of biomaterials (composition and porosity) and combined or not to PGCAP could enhance *in vivo *ectopic bone formation.

## Materials and methods

### Platelet glue obtained from cryoprecipitation of apheresis platelet products and platelet lysate preparation

**Figure 1:**
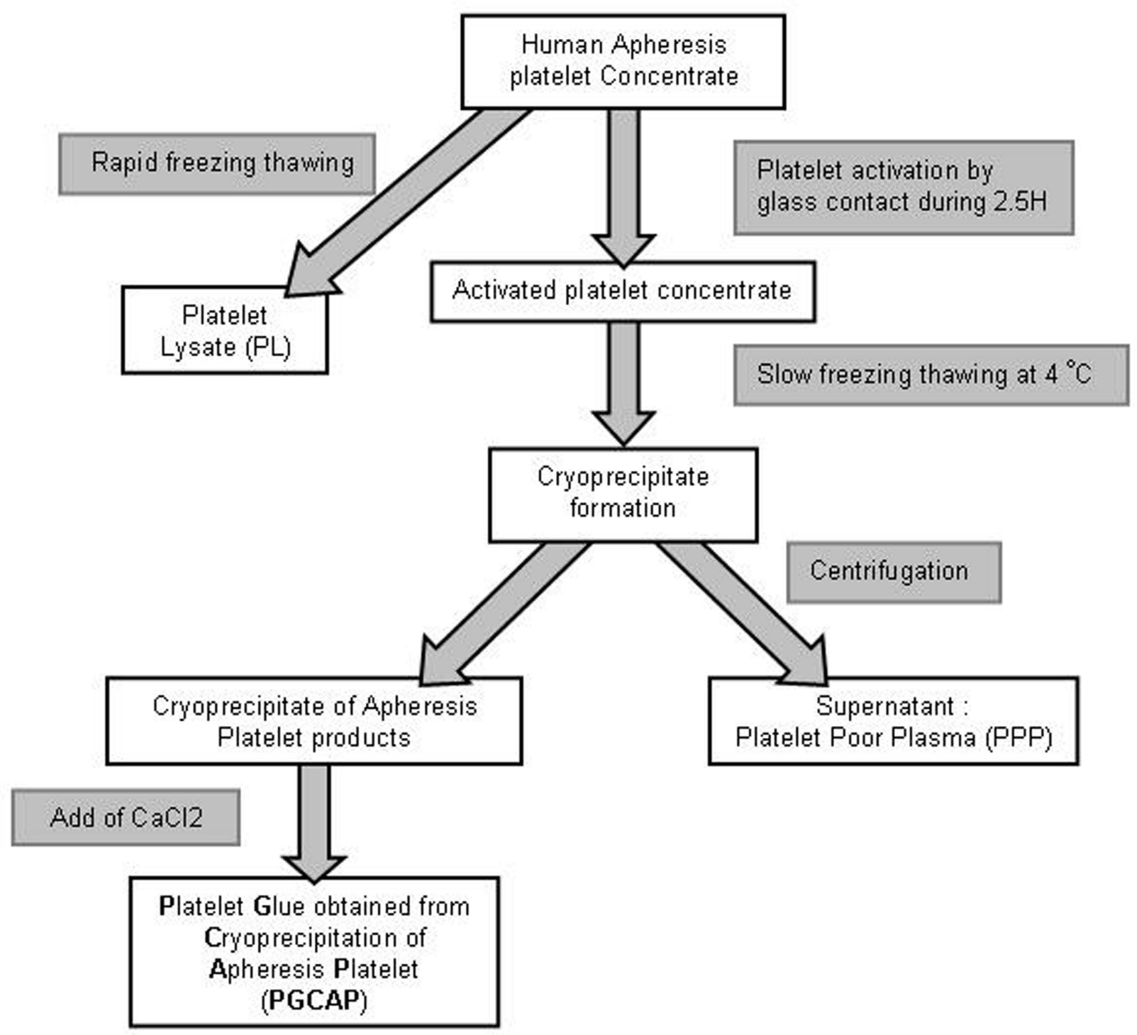
**Preparation of platelet glue obtained from cryoprecipitation of apheresis platelets**.

PGCAP and PL were made from platelet apheresis collections performed at the Centre de Transfusion Sanguine des Armées (Clamart, France). All apheresis products were biologically qualified in accordance with French legislation. Only samples containing about 1 × 10^9 ^platelets per milliliter were selected. For PL preparation, apheresis products were frozen at -40°C for lysing platelets and releasing growth factors. PL products were thawed and used in culture as fetal calf serum (FCS) substitute or for enzyme-linked immunosorbent assays (ELISAs). For PGCAP preparation (Figure 1), apheresis platelet concentrate was activated by glass contact and PGCAP was obtained through a protein cryoprecipitation process. A centrifugation was able to separate the supernatant named platelet-poor plasma (PPP) and the cryoprecipitate named PGCAP, which is composed of fibrinogen, pre-activated factors of coagulation, and platelet growth factors. For *in vitro *and *in vivo *experiments, three different mixes of PGCAP prepared from five different donors were used.

### Enzyme-linked immunosorbent assay

ELISA (Quantikine; R&D Systems, Minneapolis, MN, USA) was used to quantify the concentration of EGF (DEG00), basic FGF (HSFB75), HGF (DHG00), IGF-1 (DG100), PDGF-AB (DHB00B), TGF-β1 (DB100), VEGF (#DVE00), SDF-1α (DSA00), IL-1α (DLA50), and IL-1β (DLB50) in PGCAP, PPP, PL, Tissucol (TC) (Baxter, Deerfield, IL, USA), and FCS. All sample measurements were performed in duplicate. The lower detection limits of these assays were 0.7 pg/mL for EGF, 0.22 pg/mL for basic FGF, 40 pg/mL for HGF, 0.024 ng/mL for IGF-1, 1.7 pg/mL for PDGF-AB, 7 pg/mL for TGF-β1, 9 pg/mL for VEGF, 18 pg/mL for SDF-1α, 1 pg/mL for IL-1α, and 1 pg/mL for IL-1β. The total protein content of PGCAP, PPP, PL, TC, and FCS was determined by using Quick start Bradford dye reagent (Bio-Rad Laboratories, Inc., Hercules, CA, USA). Results were expressed as picograms of factors per 1 mg of protein ± standard error of the mean (SEM).

### MSC isolation and culture

Human bone marrow MSCs were obtained with informed consent from different patients undergoing routine total hip replacement surgery in the Percy Hospital (Clamart, France). Ethical approval for research studies on human material was given by Service de Santé des Armées (Minister of Defense). As previously reported [17], bone marrow mononuclear cells (BMMNCs) were isolated from the supernatant of spongious bone fragments, plated at a density of 100 × 10^3 ^cells/cm^2^, and cultured at 37°C in 95% air and 5% CO_2 _in alpha-minimum essential medium (α-MEM) (Biological Industries, Kibbutz Beit-Haemek, Israel) with 10 μg/mL ciprofloxacin (Ciflox 400 mg/200 mL; Bayer Pharma, Leverkusen, Germany) and 10% pre-screened FCS (FCS medium) or 5% PL (PL medium) complemented with 2 UI/mL heparin to avoid platelet gel formation [17]. After 3 to 4 days, non-adherent cells were removed and cultures were re-fed with fresh medium. Thereafter, cultures were fed at 3- to 4-day intervals. When the cells reached confluence, they were detached by using 1X trypsin-EDTA (Gibco, now part of Invitrogen Corporation, Carlsbad, CA, USA) and replated at 4,000 cells/cm^2^. For *in vivo *experiments, MSCs were transduced by lentiviral vector pRRL WPRE PGK/GFP cPPT and were purified to obtain more than 95% GFP^+ ^cells. Expanded MSCs were validated according to their classic phenotypic markers by flow cytometry, their clonogenic potential (CFU-F assay), and their multi-lineage differentiation potential (osteogenic, adipogenic, and chondrogenic) [17].

### Porous ceramic biomaterials

Calciresorb (Ceraver, Roissy, France) is a novel bone graft substitute that is used for the repair of bony voids or gaps of the skeletal system in humans. Two groups of Calciresorb biomaterials were used: (a) 'Calciresorb' biomaterials (2 to 3 mm in diameter) and (b) 'Calciresorb bone like' biomaterials (cubes of 4 × 4 × 4 mm^3^) (Table 1). In each group, three chemical compositions were evaluated: (a) beta-tricalcium phosphate (β-TCP) 35%-hydroxyapatite (HA) 65%, (b) β-TCP 75%-HA 25%, and (c) β-TCP 100% (Table 2). Before culture, biomaterials were sterilized by gamma irradiation at a minimum dose of 25 KGy. Scanning electron microscopy was used to characterize the morphology and the structure of the scaffolds.

**Table 1:** Physical characteristics of biomaterials given by Ceraver

Biomaterials	Calciresorb	Calciresorb bone like
Porosity	65% ± 5%	75% ± 5%
Pore size	100 to 400 μm	500 μm
Macroporosity	60%	100%
Microporosity	40%	-
Interconnectivity	Unknown	96% to 100%
Interconnected pore diameter	Variable	50 μm < Φ < 250 μm

**Table 2:** Chemical characteristics of biomaterials given by Ceraver

Sample list	Composition		Biomaterials
		
	HA	β-TCP	
BM_A_	65%	35%	
BM_B_	25%	75%	Calciresorb
BM_C_	-	100%	

BM_D_	65%	35%	
BM_E_	25%	75%	Calciresorb bone like
BM_F_	-	100%	

β-TCP, beta-tricalcium phosphate; BM, biomaterial; HA, hydroxyapatite.

### Preparation of MSC/biomaterial constructs

PGCAP effect was first evaluated by seeding MSCs in PGCAP or TC (27,000 cells/in products). Obtained cells/products were grown on 6-well plates. In other experiments, MSCs were loaded on biomaterials at a different cell seeding density: 1 × 10^5 ^(biocompatibility analysis) or 2 × 10^5 ^cells (for experiments that combined *in vitro *(proliferation and osteogenic differentiation studies) and *in vivo *studies). Cellular suspension (200 μL) was added on biomaterial, incubated for 3 hours, and gently agitated every 30 minutes to allow cell attachment. Before cell seeding, biomaterials were maintained in α-MEM overnight at 4°C.

For preparation of TC alone or biomaterials embedded in TC, freeze-dried human fibrinogen and thrombin were re-suspended, respectively, at 18 mg/mL and 2.5 UI/mL in 2% NaCl and 1.5 mM CaCl_2 _solution. MSCs were mixed with 100 μL of fibrinogen and with 100 μL of thrombin, immediately added on 6-well plates or biomaterials, and incubated for 15 minutes at 37°C to allow coagulation. For preparation of PGCAP alone or biomaterials embedded in PGCAP, MSCs were mixed in 200 μL of solution containing 50% PGCAP, 4.68 g/L NaCl, 0.8 g/L CaCl_2_, and 10 μg/mL exacyl [34], immediately added on wells or biomaterials, and incubated for 60 minutes at 37°C to allow coagulation. The various biomaterial constructs were then placed into 6- or 24-well culture plates in culture medium. Cell-free scaffolds were incubated under similar conditions and were used as controls.

### Quantification of cell proliferation

Cells seeded on 6-well plates or on biomaterials were rinsed with TE buffer 1x (10 mM Tris-HCl, 1 mM EDTA, pH 7.5), lysed with TE 1x 0.1% triton (Sigma-Aldrich, St. Louis, MO, USA) and 0.2 mg/mL proteinase K (Roche Applied Science, Indianapolis, IN, USA), and incubated overnight at 52°C. Then samples were frozen/thawed three times at -80°C, sonicated 15 minutes, and vortexed 1 minute. Afterward, 100 μL of lysate (with optimal dilution) was incubated in 96-well plates 10 minutes in a darkroom at room temperature with 100 μL of diluted Quant-iTTM PicoGreen double-stranded DNA (dsDNA) reagent (Molecular Probes, now part of Invitrogen Corporation), which is an ultrasensitive fluorescent nucleic acid stain for quantifying dsDNA in solution. Sample fluorescence was measured by using a fluorescence microplate reader at excitation and emission wavelengths of 485 and 535 nm, respectively, as recommended by the manufacturer. Results were compared with standard curves of series of cell dilutions (lysed as described above) and allowed us to establish a correlation between dsDNA quantity and number of cells. To exclude measurement errors, all experiments were carried out in triplicate.

### Osteogenic differentiation assays

To induce osteogenic differentiation of MSCs, the different constructs were placed in FCS medium alone (not induced-MSC = ni-MSC) or supplemented (osteogenic induced-MSC = i-MSC) with 0.1 μM dexamethasone, 0.05 mM L-ascorbic acid-2-phosphate, and 10 mM β-glycerophosphate (all from Sigma-Aldrich) for 21 days of culture. Medium was changed twice a week. For quantitative real-time reverse transcription-polymerase chain reaction (RT-PCR), on day 21, cells were lysed in RLT buffer for RNA extraction (Qiagen, Hilden, Germany). Total RNA was extracted from various MSC biomaterial constructs by using RNeasy a micro- or mini-kit as described by the manufacturer (Qiagen) and treated with DNAse (Roche, Basel, Switzerland). cDNA was synthesized from 0.5 μg of total RNA by using Random Hexamer Primer and Multiscribe RT (Applied Biosystems, Foster City, CA, USA). Gene expression was quantified by real-time PCR by using the LightCycler Fast Start DNA Master (Roche) with 0.2 μL of cDNA corresponding to 2 ng of total RNA in a 20-μL final volume, 2.25 mM MgCl_2_, and 0.5 μM of each primer. PCR was performed for 45 cycles at 95°C for 15 seconds, at the specific annealing temperature for 25 seconds, and at 72°C for 30 seconds. Amplification specificity was checked by using a melting curve. Specific gene primers for each factor of interest were designed for real-time PCR analysis by using Primer3 and Ensembl software (Table 3). Results were analyzed with LightCycler software version 3.5 (Roche) by using the second derivative maximum method to set the threshold cycle (C_T_). Quantitative analysis was carried out by using standard curves and normalization with the GeNorrm software and methodology [35], with four reference genes: glyceraldehyde-3-phosphate dehydrogenase (*GAPDH*), β-actin, β-2-microglobulin, and hypoxanthine phosphoribosyltransferase 1 (*HPRT*).

**Table 3:** Polymerase chain reaction primers used to investigate osteoblastic cell differentiation

Gene	Identification	Forward primer	Reverse primer
Alkaline phosphatase	NM_000478	GGCCCTACAATGCTCATGTC	TGGTGGTCTTGGAGTGAGTG
Collagen type I, alpha 1	NM_000088	GAATGGAGATGATGGGGAAG	CCATCCAAACCACTGAAACC
Osteocalcin (*Bglap*)	NM_199173	GACTGTGACGAGTTGGCTGA	GCAAGGGGAAGAGGAAAGAA
Osteonectin (*Sparc*)	NM_003118	GGGCTTCTCCTCCTCTGTCT	AACCGATTCACCAACTCCAC
Osteopontin (*Spp1*)	NM_001040058	GCCGAGGTGATAGTGTGGTT	CATTCAACTCCTCGCTTTCC
Runx 2	NM_001024630	GCACTGGGTCATGTGTTTGA	GGCTGCATTGAAAAGACTGC
β-Actin	NM_001101	TCGTGCGTGACATTAAGGAG	AGGAAGGAAGGCTGGAAGAG
β-2-microglobulin	NM_004048	TGTCTTTCAGCAAGGACTGG	CCTCCATGATGCTGCTTACA
*Gapdh*	NM_002046	CCAGGTGGTCTCCTCTGACT	GGTGGTCCAGGGGTCTTACT
*Hprt1*	NM_000194	GACCAGTCAACAGGGGACAT	CTTGCGACCTTGACCATCTT

*Gapdh*, glyceraldehyde-3-phosphate dehydrogenase; *hprt1*, hypoxanthine phosphoribosyltransferase 1.

### Animal model

Thirty Swiss nude mice (6 weeks old; Charles River Laboratories, Inc., Wilmington, MA, USA) were used in these experiments, kept in a controlled environment, and treated in accordance with the institutional animal guidelines. MSCs loaded on BM_A _or BM_F _embedded or not in PGCAP were cultured in the presence or absence of osteogenic induction medium for 21 days. On each mouse, four implants per condition were grafted. Avertine was used for anesthesia to proceed to incisions on a 5-mm length to create subcutaneous pockets. Biomaterials were implanted in the upper part of the back, and next the skin was closed by suture with non-absorbable thread. After 28 days, the animals were euthanized by inhalation of carbon dioxide. The samples were harvested, washed once in PBS, and immediately put in paraformaldehyde (PFA) 4% (confocal microscopy analysis) or in formol fixative (histology analysis). This protocol was approved by the ethics committee for animal experiments of the Army Biomedical Research Institute of Bretigny-sur-Orge.

### Confocal microscopy

Adhesion, proliferation, and persistence of MSCs on various biomaterials were assessed by using confocal microscopy. The cell-seeded scaffolds were cut in four slices and fixed with PFA 4% for 60 minutes at room temperature. Fixed samples were rinsed with PBS and incubated with blocking buffer (PBS, 2% human albumin, and 5 mg/mL human immunoglobulin; LFB, Les Ulis, France) for 1 hour at 4°C, followed by overnight incubation with the primary antibody (IgG1-APC or CD90-APC; Beckman Coulter, Inc., Fullerton, CA, USA) at 4°C in blocking buffer. After washing with PBS, samples were counterstained with 4'-6-diamidino-2-phenylindole (DAPI) (Sigma-Aldrich) at 300 ng/mL for 1 hour. Images were obtained and processed by using a TCS SPE confocal microscope (Leica, Wetzlar, Germany) with a ×20 objective (numerical aperture of 0.75). The excitation lasers used were 405 nm to image nuclei stained with DAPI and 488, 532, and 635 nm to image MSC GFP^+^, Biomaterial (reflectance), and CD90APC, respectively.

### Histology and immunohistochemistry

Implanted biomaterials were rinsed and immersed in formol for 1 day. The various biomaterials were decalcified with 0.1 M EDTA 2Na for 3 to 4 days (Calciresorb biomaterials) or with 0.6 M EDTA 2Na for 14 to 21 days (Calciresorb bone like biomaterials). Biomaterials were rinsed with PBS, re-fixed with formol, and then dehydrated with a graded series of ethanol treatment prior to being embedded in paraffin. Paraffin sections of 5-μm thickness were dried, deparaffinized, and stained with hematoxylin, phloxin, and safranin or treated for immunohistochemistry.

For collagen immunohistological staining [36], sections were pre-treated with 2 mg/mL hyaluronidase (Merck, Whitehouse Station, NJ, USA) for 15 minutes at 37°C and subsequently with 1 mg/mL pronase (Sigma-Aldrich) for 30 minutes at 37°C. Endogenous peroxidases were blocked by using 3% H_2_O_2 _for 5 minutes. Non-specific background was blocked by using TBS1X containing 10% FBS for 45 minutes. Sections were incubated overnight at 4°C with monoclonal mouse anti-human type I collagen (clone I-8H5, 1 μg/mL; Acris Antibodies, San Diego, CA, USA). The Envision^™ ^FLEX^+ ^(Dako, Glostrup, Denmark) with Dako autostainer instrument was then used for staining analysis.

Bone formation area was quantified by using stained histological sections. Images were analyzed with Photoshop and ImageJ software. The ratio of all bone formation area on total biomaterial area was measured on three to five sections at different levels of the biomaterial (near the surface and in the center of biomaterial).

### Statistical analysis

For cell growth analysis, all measurements were performed on three different sources of MSCs and three different batches of PGCAP. *In vivo *experiments were performed in triplicate. All data are expressed as mean ± SEM. Statistical comparisons were made by using analysis of variance (ANOVA) in which a *P *value of less than 0.05 was considered significant. Differences between groups or individual conditions were evaluated by using Student Newman-Keuls test.

## Results

### PGCAP is enriched in growth factors and SDF-1α and promotes MSC and osteoprogentitor proliferation

We aimed to determine whether PGCAP was enriched in growth factors involved in MSC proliferation and bone repair. For this purpose, we first quantified growth factor, cytokine, and chemokine contents of PGCAP, PPP, PL, TC, and FCS by ELISA. Our results showed that EGF, FGF-β, HGF, PDGF-AB, TGF-β1, and VEGF concentrations were significantly higher in PGCAP than in PPP (3.65 -, 3.04-, 1.74-, 3.38-, 138.4-, and 6.03- fold, respectively) and in PL (6.52-, 2.41-, 1.13-, 10.28-, 1.84-, and 5.83-fold, respectively) (Table 4). Few factors were detected in TC and FCS as compared with PGCAP (Table 4), according to their plasmatic origin. We also found that chemokine SDF-1α concentration was significantly higher in PGCAP than in PPP (5.69-fold), PL (3.9-fold), TC (2.19-fold), and FCS (3.8-fold). In contrast, IGF-1 concentration was slightly higher in PPP and PL than in PGCAP. Few concentrations of IL-1α and IL-1β were detected in PGCAP, although IL-1β was slightly higher in PGCAP than in PPP (Table 4). We further showed that fibrinogen was highly concentrated in PGCAP as compared with PPP and was similar to TC (data not shown). Together, these results suggest that the cryoprecipitation process allows concentrating plasma and platelet-related molecules such as fibrinogen, growth factors, and SDF-1α.

**Table 4:** Enzyme-linked immunosorbent assay quantitation of growth factor, cytokine, and chemokine levels in PGCAP, PPP, PL, TC, and FCS

		EGF	FGF-b	HGF	IGF-1	PDGF-AB	TGF-b	VEGF	SDF-1a	IL-1a	IL-1b
Picograms per 1 mg protein, mean ± SEM	PGCAP	289.87 ± 50.14	5.36 ± 0.62	21.24 ± 2.73	609.61 ± 81.92	7,249.57 ± 1,305.81	2,166.77 ± 248.38	99.55 ± 26	142.88 ± 7.87	0.07 ± 0.01	2.60 ± 0.99
	
	PPP	79.36 ± 7.93	1.76 ± 0.16	12.19 ± 1.1	3,310.22 ± 362.1	2,146.71 ± 237.89	15.66 ± 1.81	16.50 ± 2.08	25.10 ± 2.34	0.19 ± 0.03	0.35 ± 0.20
	
	PL	44.48 ± 2.09	2.22 ± 0.91	18.76 ± 1.76	2,622.15 ± 279.86	705.42 ± 84.94	1,180.52 ± 37.61	17.09 ± 3.08	36.67 ± 5.46	0.10 ± 0.02	0.23 ± 0.05
	
	TC	N.D.	0.04	0.07	16.62	0.03	42.91	0.13	65.22	0.23	0.30
	
	FCS	N.D.	0.11	N.D.	1,916.29 ± 27.95	N.D.	100.03	N.D.	37.60 ± 1.22	0.57 ± 0.32	0.07

Picograms for 100 μL of products	PGCAP	2,683.15 ± 325.20	50.26 ± 6.45	189.57 ± 19.91	5,341.28 ± 395.90	63,806.01 ± 5,642.06	21,407.23 ± 2,732.49	962.80 ± 83.56	926.25 ± 84.40	0.41 ± 0.08	24.89 ± 12.83

	TC	n.d.	0.50	0.84	191.08	0.31	493.50	1.50	150.00	0.53	0.68

Ratio	PGCAP/ PPP	3.65^a^	3.04^b^	1.74^c^	0.18 ^N.S.^	3.38^d^	138.36^a^	6.03^a^	5.69^b^	0.38 ^N.S.^	7.37^c^
	
	PGCAP/ PL	6.52^a^	2.41^b^	1.13 ^N.S.^	0.23 ^N.S.^	10.28^d^	1.84^a^	5.83^a^	3.9^b^	0.71 ^N.S.^	11.23 ^N.S.^
	
	PGACP/TC	N.D.	123.31^c^	292.51 ^N.S.^	36.68 ^N.D.^	269,806.08^a^	50.49^d^	763.24^a^	2.19^a^	0.31 ^N.S.^	8.77 ^N.S.^
	
	PGCAP/FCS	N.D.	50.93^c^	N.D.	0.32^c^	N.D.	21.66^d^	N.D.	3.8^a^	0.12^c^	36.24 ^N.S.^
	
	PPP/PGCAP	0.27 ^N.S.^	0.33 ^N.S.^	0.57 ^N.S.^	5.43^d^	0.3 ^N.S.^	0.01 ^N.S.^	0.17 ^N.S.^	0.17 ^N.S^.	2.62 ^N.S.^	0.14 ^N.S.^
	
	PL/PGCAP	0.15 ^N.S.^	0.41 ^N.S.^	0.88 ^N.S.^	4.3^d^	0.1 ^N.S.^	0.54 ^N.S.^	0.17 ^N.S.^	0.26 ^N.S.^	1.41 ^N.S.^	0.09 ^N.S.^

Analysis-of-variance test followed by Student Newman-Keuls test. ^a^*P *<0.001, ^b^*P *<0.01, ^c^*P *<0.05, ^d^*P *<0.0001. EGF, epidermal growth factor; FCS, fetal calf serum; FGF-b, fibroblast growth factor-beta; HGF, hepatocyte growth factor; IGF-1, insulin-like growth factor-1; IL, interleukin; N.D., not determined; N.S., not significant; PDGF-AB, platelet-derived growth factor-AB; PGCAP, platelet glue obtained from cryoprecipitation of apheresis platelet products; PL, platelet lysate; PPP, platelet-poor plasma; SDF-1a, stromal cell-derived factor 1 alpha; SEM, standard error of the mean; TC, Tissucol; TGF-b, transforming growth factor-beta; VEGF, vascular endothelial growth factor.

The influence of PGCAP on MSC phenotype was investigated at each culture passage (from P0 to P3) by flow cytometry. We showed that MSC phenotypic profile was unchanged regardless of the culture condition since more than 98% of expanded MSCs were positive for CD90, CD105, and CD73 and were negative for CD45 (data not shown).

**Figure 2:**
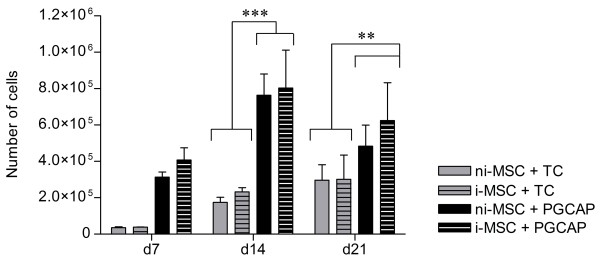
**PGCAP promotes mesenchymal stromal cell (MSC) and osteoprogenitor proliferation**. Not induced-MSCs (ni-MSCs) or osteogenic induced-MSCs (i-MSCs) were embedded in TC or PGCAP and grown for 21 days. The number of cells was evaluated in each condition at days 7, 10, and 21. Data are expressed as mean ± standard error of the mean. Analysis-of-variance test followed by Student Newman-Keuls test revealed statistically significant difference between groups (PGCAP and TC) at different times. **P <0.01, ***P <0.001. d, day; PGCAP, platelet glue obtained from cryoprecipitation of apheresis platelet products; TC, Tissucol.

As indicated by results from Table 4, a higher amount of growth factors was present in PGCAP than in TC products. Therefore, we further studied the impact of PGCAP or TC on MSC proliferation at different times of osteoblastic differentiation (Figure 2). ni-MSC and i-MSC proliferation was highly enhanced in PGCAP as compared with TC condition at day 14 (3.84-fold, *P *<0.001) and at day 21 (1.85-fold, *P *<0.01). These results suggest that PGCAP enhances proliferation of both MSCs and osteoprogenitor-induced MSCs.

### Physical characteristics and biocompatibility of biomaterials

**Figure 3:**
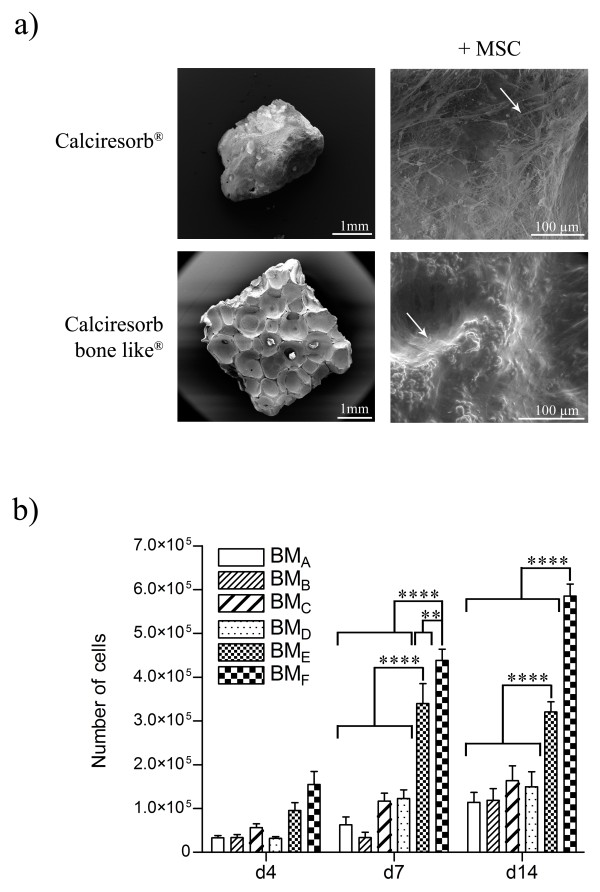
**Physical characteristics and biocompatibility of biomaterials and mesenchymal stromal cell (MSC) proliferation on the different biomaterials**. **(a) **Representative scanning electron microscopy images of 'Calciresorb' (BM_A_) and 'Calciresorb bone like' (BM_F_) biomaterial surfaces are shown. MSCs were loaded on these two biomaterials and grown in platelet lysate (PL) medium. MSC colonization on the various biomaterials was evaluated by scanning electron microscopy at 14 days of culture. Arrows indicate cells on biomaterials. **(b) **MSCs loaded on the various biomaterials at low cell seeding density (100 × 10^3 ^cells per biomaterial) were grown in PL medium for 14 days. The number of cells on the different biomaterials was evaluated at days 4, 7, and 14. The data are expressed as mean ± standard error of the mean. A statistically significant difference between individual conditions was revealed by analysis-of-variance test followed by Student Newman-Keuls test. **P <0.01, ****P <0.0001. d, day.

Two different biomaterials (Table 1) and three different compositions (Table 2) of biomaterials were analyzed by scanning electron microscopy. Pictures shown in Figure 3a illustrate data obtained from Ceraver. To evaluate the biocompatibility of the various biomaterials selected, MSCs were seeded on each biomaterial and cultured for further analysis by scanning electron microscopy and for proliferation evaluation. As shown in Figure 3a, the pores of each type of biomaterial were colonized by MSCs. This suggests that the chemical composition or structure of biomaterials did not interfere with the cell colonization.

A kinetic study of MSC proliferation was performed by using the different biomaterials (Figure 3b). At days 7 and 14 of culture, BM_F _allowed significantly higher MSC proliferation than the other biomaterials tested (Figure 3b). Even if BM_E _was less powerful than BM_F_, we showed that BM_E _allowed a better MSC proliferation than BM_A_, BM_B_, BM_C_, and BM_D _at days 7 and 14. Between days 0 and 4, the number of cells did not vary between each condition (data not shown). These results indicate that the structure of Calciresorb bone like biomaterial combined with a 100% β-TCP chemical composition improves MSC proliferation. Considering these results, we decided to use the BM_F _for further experiments in comparison with the BM_A_, which is widely studied and reported in the literature.

### PGCAP improves proliferation of cells loaded on BM_A_

**Figure 4:**
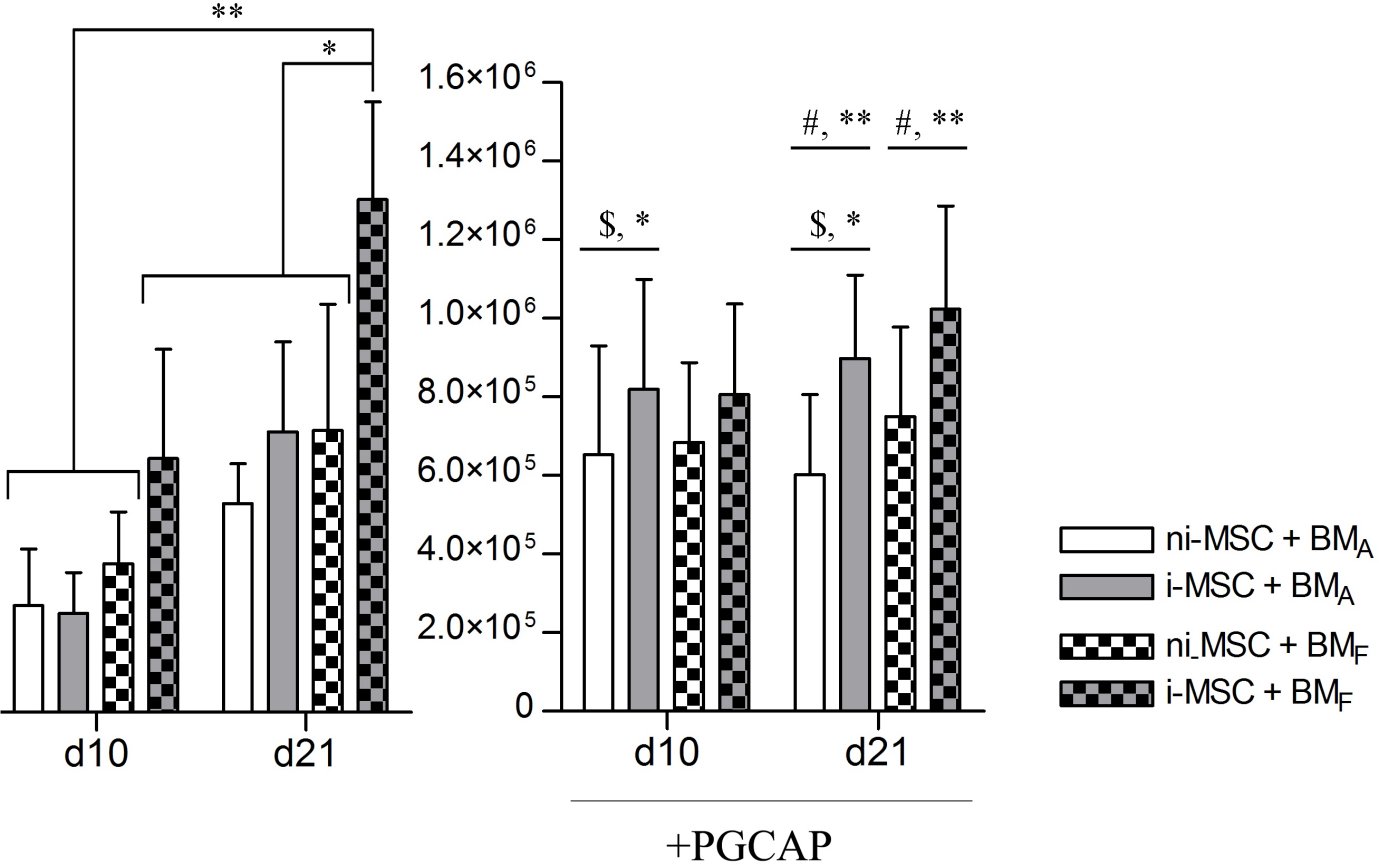
**Effect of PGCAP on mesenchymal stromal cell (MSC) and osteoprogenitor proliferation loaded on BM_A _and BM_F_**. Not induced-MSCs (ni-MSCs) or osteogenic induced-MSCs (i-MSCs) loaded on BM_A _and BM_F _biomaterials at high cell seeding density (20 × 10^3 ^cells per biomaterial) were embedded or not in PGCAP and grown for 21 days. The number of cells was evaluated in each condition at days 10 and 21. The data are expressed as mean ± standard error of the mean. A statistically significant difference between individual conditions (without PGCAP) was revealed by analysis-of-variance test followed by Student Newman-Keuls. *P <0.05, **P <0.01. With PGCAP, similar tests revealed statistically significant difference between group comparing ni-MSCs and i-MSCs loaded on BM_A _(^$^*P <0.05) or group comparing ni-MSCs and i-MSCs at day 21 (^#^**P <0.01). PGCAP, platelet glue obtained from cryoprecipitation of apheresis platelet products.

We further investigated the influence of PGCAP in the presence or absence of osteogenic inductors on proliferation of MSCs loaded on biomaterials. A kinetic proliferation study of ni-MSCs and i-MSCs loaded on BM_A _and BM_F _embedded or not in PGCAP was performed at days 10 and 21. Without PGCAP, we showed that the proliferation of i-MSCs loaded on BM_F _was significantly higher at day 21 than that of ni-MSCs (*P *<0.05) (Figure 4, left panel). Furthermore, we showed that the proliferation of i-MSCs loaded on BM_F _was significantly higher at day 21 than at day 10 (*P *<0.05). Therefore, i-MSC proliferation was significantly higher when loaded on BM_F _than on BM_A _at day 21 (*P *<0.05) (Figure 4, left panel). Interestingly, ni-MSC proliferation was not improved when loaded on BM_F _as compared with BM_A_, which is contradictory to our previous results shown in Figure 3b. In fact, owing to a higher cell seeding density (200 × 10^3 ^versus 100 × 10^3 ^cells per biomaterial), cell proliferation reaches a plateau and a difference between the two biomaterials could not be seen.

In the presence of PGCAP, we showed that osteogenic induction enhanced the proliferation of MSCs loaded on BM_A _embedded in PGCAP regardless of the day of analysis (^$^*P *<0.05, Figure 4, right panel). At day 21, the proliferation of i-MSCs embedded in PGCAP was significantly higher as compared with ni-MSCs regardless of the biomaterial tested (^#^*P *<0.05, Figure 4, right panel). Furthermore, we showed that PGCAP enhanced proliferation of ni-MSCs and i-MSCs loaded on BM_A _(*P *<0.05, Figure 4). These results suggest that PGCAP is able to improve cell proliferation on BM_A _(which has a poor proliferative potential) but that its proliferative effect is less effective on BM_F _(which has a high proliferative potential).

### PGCAP improves osteogenic differentiation of MSCs loaded on biomaterials

**Figure 5:**
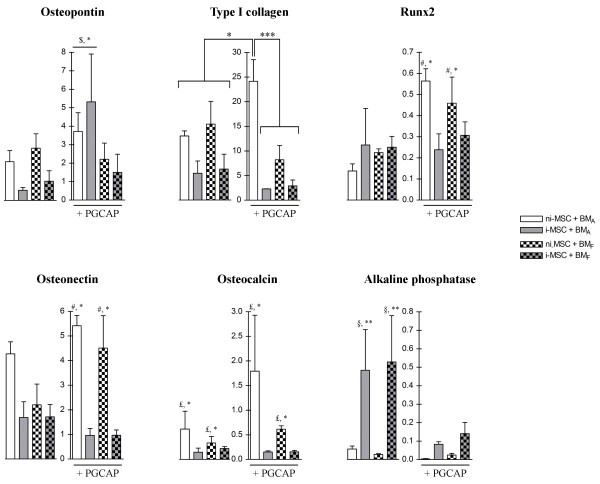
**PGCAP improves osteogenic differentiation of mesenchymal stromal cells (MSCs) loaded on biomaterials**. Total RNAs were extracted from ni-MSCs and i-MSCs loaded on BM_A _and BM_F _at high cell seeding density (200 × 10^3 ^cells per biomaterial) embedded or not in PGCAP and grown for 21 days. Quantifications of osteopontin, type I collagen, Runx2, osteonectin, osteocalcin, and alkaline phosphatase mRNA expression were analyzed by real-time quantitative-polymerase chain reaction. Transcript levels in arbitrary units are expressed as mean ± standard error of the mean. A statistically significant difference between individual conditions or groups of condition was revealed by analysis-of-variance test followed by Student Newman-Keuls test. Osteopontin: group of ni-MSCs and i-MSCs loaded on BM_A _embedded in PGCAP was compared with other conditions (^$^*P <0.05). Type I collagen: individual comparisons, *P <0.05, **P <0.01, ***P <0.001. Runx2, Osteonectin: group with ni-MSCs embedded in PGCAP (loaded in both biomaterials) was compared with other conditions with i-MSCs (^#^*P <0.05). Osteocalcin: comparison of group osteogenic-induced or not (^£^*P <0.05). Alkaline phosphatase: group with i-MSCs without PGCAP (loaded in both biomaterials) was compared with other conditions with ni-MSCs (^§^**P <0.01). i-MSC, not osteogenic induced-mesenchymal stromal cell; ni-MSC, not induced-mesenchymal stromal cell; PGCAP, platelet glue obtained from cryoprecipitation of apheresis platelet products.

The influence of PGCAP in the presence or absence of osteogenic inductors, on osteogenic differentiation potential of MSCs loaded on BM_A _and BM_F_, was next investigated by osteoblastic gene expression analysis at day 21 of osteogenic induction (Figure 5). The role of *in vitro *osteogenic induction of MSCs on the mRNA expression of osteogenic markers was further analyzed. Our results showed that alkaline phosphatase mRNA expression was upregulated by osteogenic induction in the absence of PGCAP regardless of the biomaterial tested (12.2-fold, ^§^*P *<0.01; Figure 5). These results obtained here correlated with results from alkaline phosphatase activity study, showing that, in the presence of osteogenic inductors, cells exhibit alkaline phosphatase activity on both biomaterials (data not shown). In MSCs loaded on both types of biomaterials, osteogenic induction downregulated the expression of osteonectin (5.13-fold, ^#^*P *<0.001) and Runx2 (1.83-fold, ^#^*P *<0.05) in the presence of PGCAP. Osteogenic induction also downregulated the expression of osteocalcin mRNA (7.88-fold, ^£^*P *<0.05) in the presence or absence of PGCAP (Figure 5). Type I collagen mRNA expression was downregulated by osteogenic induction medium (10.55-fold, *P *<0.01) only in MSCs loaded on BM_A _embedded in PGCAP (Figure 5).

Then our results showed that PGCAP enhanced the osteopontin mRNA expression in ni-MSCs and i-MSCs loaded on BM_A _(^$^3.26-fold, *P *<0.05; Figure 5). PGCAP upregulated the collagen type I mRNA expression in MSCs loaded on BM_A _only with ni-MSCs (1.85-fold, *P *<0.05; Figure 5). We also showed that PGCAP upregulated Runx2 and osteonectin mRNA expression only in ni-MSCs regardless of the biomaterials (2.82- and 1.52-fold, respectively, *P *<0.05; Figure 5). Surprisingly, alkaline phosphatase mRNA expression was decreased by PGCAP in MSCs loaded on both biomaterials in the presence or absence of osteogenic inductors (4.33-fold, *P *<0.01), suggesting that PGCAP could contain high levels of osteogenic inductors that downregulate alkaline phosphatase mRNA expression. Therefore, we demonstrate that PGCAP stimulates by itself the osteogenic differentiation of MSCs as ascertained by its capacity to upregulate the mRNA expression of osteogenic markers (collagen type I, osteonectin, osteopontin, and Runx2).

### Ectopic implantation of MSC-loaded biomaterials embedded in PGCAP promotes bone formation in nude mice

**Figure 6:**
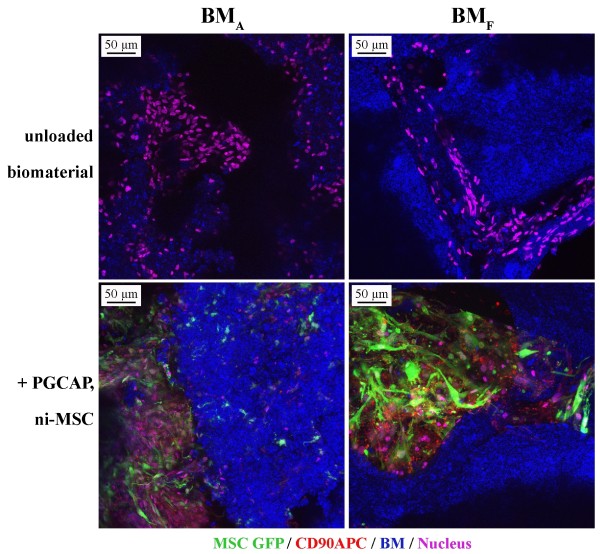
***Ex vivo *confocal microscopy analysis of BM_A _and BM_F _biomaterials**. Not induced- mesenchymal stromal cells (ni-MSCs) or osteogenic induced-MSCs (i-MSCs) GFP^+ ^loaded on BM_A _and BM_F _at high cell seeding density (200 × 10^3 ^cells per biomaterial) were embedded or not in PGCAP and grown for 21 days. Next, biomaterials were subcutaneously grafted on nude mice. Unloaded biomaterials (no pre-loaded cells and not embedded in PGCAP) were also grafted on mice. Twenty-eight days after grafting, biomaterials were cut in four pieces, fixed, and stained with CD90 antibody. Biomaterials were analyzed by confocal microscopy (blue: biomaterial; green: MSC GFP^+^; red: CD90APC; and purple: DAPI). Original magnification: ×20. Each picture is representative of three independent experiments. DAPI, 4'-6-diamidino-2-phenylindole; PGCAP, platelet glue obtained from cryoprecipitation of apheresis platelet products.

Bone formation ability of MSC-loaded biomaterials embedded in PGCAP was investigated after ectopic implantation in a nude mouse model. BM_A _and BM_F _were tested in combination with ni-MSCs versus i-MSCs, in the presence or absence of PGCAP. Four weeks after implantation, biomaterials were analyzed *ex vivo *by confocal microscopy (Figure 6). When unloaded biomaterials were implanted, we observed mouse cell invasion inside both BM_A _and BM_F_, suggesting that biomaterial implantation induced host cell recruitment. We then tested biomaterials in the presence of GFP^+ ^ni-MSCs or i-MSCs after 21-day culture in the presence or absence of PGCAP. We first demonstrated that MSCs remain GFP^+ ^after 21-day culture (data not shown). When these MSC-loaded biomaterials were implanted in mice, GFP^+^/CD90^+ ^cells were observed inside the pores of all types of biomaterials and inside PGCAP (Figure 6). These results suggest that, after 4-week implantation, MSCs survived in the host independently of the various tested conditions.

**Figure 7:**
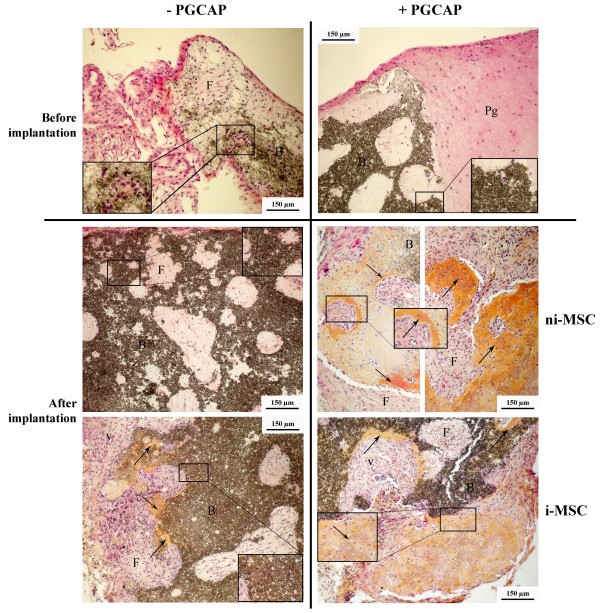
**Ectopic implantation of MSC-loaded BM_A _embedded in PGCAP promotes bone formation in nude mice**. Not induced-MSCs (ni-MSCs) or osteogenic induced-MSCs (i-MSCs) GFP^+ ^loaded on BM_A _at high cell seeding density (200 × 10^3 ^cells per biomaterial) were embedded or not in PGCAP and grown for 21 days. Next, biomaterials were grafted on nude mice. After 21 days of *in vitro *culture (before implantation) and 28 days after grafting (after implantation), biomaterials were decalcified, embedded in paraffin, and stained with hematoxylin (nuclei), phloxin (cytoplasm), and safranin (matrix). Original magnification: ×10. Boxed area is magnified and points out cells that have migrated in biomaterials or area of bone formation. Arrows represent bone-like tissue characterized by osteocyte-like cells embedded in matrix stained by safranin. Each picture is representative of three independent experiments. B, biomaterial; F, fibroid tissue; Pg, platelet glue obtained from cryoprecipitation of apheresis platelet products; PGCAP, platelet glue obtained from cryoprecipitation of apheresis platelet products; V, vessels.

We then performed histological analysis on decalcified biomaterials before and after 4-week implantation. We showed that fibroblast-like cells were homogenously detected in PGCAP and BM_A _(Figure 7) or BM_F _(data not shown) pores just before implantation (see magnified views of the boxed areas). After the implantation period (Figure 7), vessels were observed in all biomaterials, suggesting that vascularization induction was not dependent on the presence of PGCAP and osteogenic induction. We observed that, after 4-week implantation, the biomaterial exhibits large bone-like tissue zones with some of the characteristics of woven bone (in pores and PGCAP) when it was embedded in PGCAP and loaded with ni-MSCs or i-MSCs (Figure 7). More bone-like tissue zones were observed near the surface than in the center of the biomaterial. When biomaterials loaded with ni-MSCs were implanted in the absence of PGCAP, we observed mainly areas of fibroid tissue. Conversely, we observed few bone-like tissue zones when the implanted biomaterials were loaded with i-MSCs in the absence of PGCAP.

**Figure 8:**
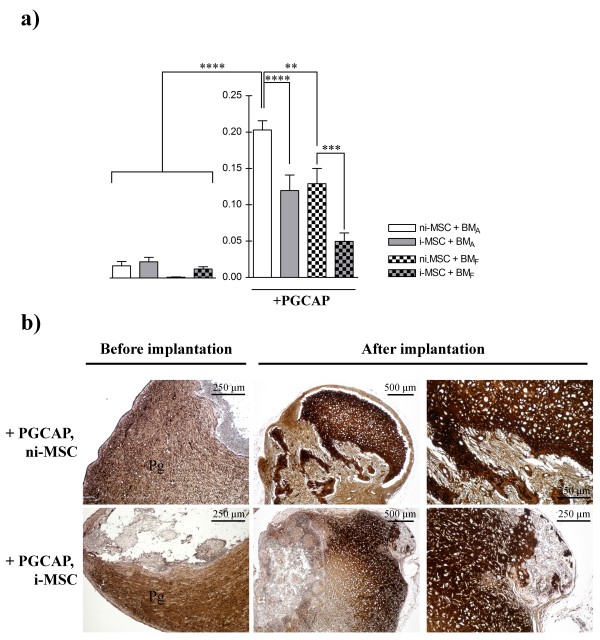
**Bone formation areas are increased after ectopic implantation of MSC-loaded BM_A _embedded in PGCAP**. **(a) **The average ratio of bone formation area in BM_A _28 days after grafting was determined in each condition. The data are expressed as mean ± standard error of the mean. A statistically significant difference between individual conditions was revealed by analysis-of-variance test followed by Student Newman-Keuls test. *P <0.05, **P <0.01, ***P <0.00, ****P <0.0001. **(b) **Type I collagen immuno-staining (brownish color) was performed on BM_A _and BM_F _sections before implantation and 28 days after implantation. Original magnifications: ×4 and ×10. i-MSC, not osteogenic induced-mesenchymal stromal cell; MSC, mesenchymal stromal cell; ni-MSC, not induced-mesenchymal stromal cell; PGCAP, platelet glue obtained from cryoprecipitation of apheresis platelet products.

Quantification of bone-like tissue areas revealed that PGCAP allowed higher bone formation in ni-MSC-loaded biomaterials (12-fold for BM_A_, *P *<0.0001, and 106-fold for BM_F_, *P *<0.001; Figure 8a). Our result also showed that osteogenic induction decreased bone-like tissue formation area in biomaterial loaded by MSCs and embedded in PGCAP (1.7-fold for BM_A_, *P *<0.0001, and 2.6-fold for BM_F_, *P *<0.001; Figure 8a). Furthermore, bone-like tissue formation areas in BM_A _loaded with ni-MSCs and embedded in PGCAP were significantly higher than in BM_F _(1.57-fold, *P *<0.01; Figure 8a).

Type I collagen immunohistochemical staining sections were performed on BM_A _and BM_F _before and after implantation in mice. Before implantation, type I collagen staining was limited to PGCAP area (Figure 8b). After 4-week implantation, type I collagen staining was observed in biomaterial pores and in PGCAP areas corresponding to the previously described bone-like tissue zones. Furthermore, a higher type I collagen staining was observed in biomaterials when they were embedded in PGCAP as compared with the conditions in the absence of PGCAP (data not shown). Together, our results demonstrate that BM_A _was more efficient at promoting bone-like tissue formation and suggest that the addition of PGCAP leads to a higher stimulation of bone-like tissue formation as compared with osteogenic induction of MSCs.

## Discussion

In recent years, the search for a substitute to cancellous bone autograft in large bone defects has focused on composite materials. Several parameters such as the nature of the conductive material, differentiation state of implanted cells, and the local supplementation in growth factors have been shown to be crucial in bone repair. Owing to the number of parameters to be tested, their relative importance and interactions still require further basic research. We focused our study on a blood-derived product as an easily available source of clinical-grade growth factors: a platelet glue obtained from cryoprecipitation of apheresis platelet products (PGCAP). We showed that, *in vitro*, PGCAP enhanced both proliferation and osteoblastic differentiation of bone marrow-derived MSCs. We confirmed these results by demonstrating the ability of PGCAP to improve ectopic bone formation in mice after implantation of MSC-loaded biphasic biomaterials. Interestingly, we showed that this approach was more efficient than using MSC-derived osteoprogenitor cells obtained after an *in vitro *osteoblastic induction of MSCs.

Several teams have worked on platelet-derived product optimization processes which led to various acronyms such as (a) PRP [37], (b) concentrated PRP (cPRP) [38], (c) 'thorn PRP' [39], and (d) 'Choukroun platelet-rich fibrin' (PRF) [40]. Other studies reported that freezing/thawing procedures allowed platelet lysate (PL) [17] or cryoprecipitate (Cryo) [41] to be obtained. In our process of PGCAP preparation, platelets were activated on a glass support, leading to the initiation of coagulation without requiring exogenous factors [42]. Then platelets were successively frozen/thawed, allowing arrest of the coagulation cascade, cryoprecipitation of plasmatic proteins (such as fibrinogen). Platelet lysis induced by the process allowed delivering platelet growth factors in the PGCAP. As shown in our results, the PGCAP preparation process allowed higher growth factor and cytokine concentrations than other tested products (PPP, PL, TC, and FCS). Moreover, Bieback and colleagues [43] showed that PL was more powerful to enhance MSC proliferation, while keeping their immune and differentiation potential, as compared with human serum or thrombin-activated PRP. PGCAP was obtained from apheresis products which contain 1 × 10^9 ^platelets per milliliter and this is in agreement with the platelet concentration used by Weibrich and colleagues [44] for bone repair. It is important to note that our cryoprecipitation process leads to a high growth factor concentration that does not alter MSC and osteoprogenitor phenotype and their *in vivo *capacity to form ectopic bone. Furthermore, in our process, gel formation was obtained by using endogenous fibrinogen and thrombin without the addition of several chemical molecules [39] or xenogenic thrombin, that could be of interest for a clinical application. Together, these results confirmed the advantage of platelet lysis combined with cryoprecipitation process to deliver growth factors in the final product as compared with the other platelet-derived products described above.

In addition to having a function in hemostasis, platelets play an important role in inflammation and tissue wound repair through paracrine pathways or cell-cell interactions [45]. Upon activation, platelets release a wide range of factors that are involved in bone repair by promoting survival and proliferation of various cells (fibroblasts, MSCs, endothelial cells, osteoblasts, and chondrocytes) [17,22]. Furthermore, some of these growth factors (EGF, FGF, IGF-1, and SDF-1α) have been shown to induce MSC osteoblastic differentiation [46] whereas others (PDGF, TGF-β, and HGF) exhibit controversial effects on osteogenesis depending or not on MSC differentiation stages [47,48]. VEGF plays an essential role in angiogenesis [49], and several studies have investigated its role in the enhancement of fracture healing and non-unions [50]. Moreover, these factors released by platelets have chemotactic effects on various cell types [51]. Thus, all of these observations support the idea that platelet factors exhibit various effects promoting bone regeneration.

The physical and chemical properties of biomaterials are known to influence proliferation and differentiation of MSCs and osteoformation *in vivo*. These parameters also act on inflammatory reactions within the implantation bed. Malard and colleagues [52] suggested that a strong but brief inflammatory reaction associated with microparticles, which implies a massive release of cytokines, was favorable to the bone-healing process. This finding emphasized the importance of host response to tissue-engineering devices [53]. In our study, moderate inflammation was observed but no significant differences were obtained with our various conditions. This indicates that the difference of structure between Calciresorb and Calciresorb bone like biomaterials did not seem to influence this parameter.

The geometry and macrostructural properties of biomaterials have been shown to play important roles: to supply nutrients and oxygen, to allow infiltration of cells and tissue, and to provide pores, channels, concavities, or spaces in which processes leading to heterotopic bone formation can occur without being disturbed by high body fluid refreshments or mechanical forces. In our study, confocal and histological analyses have shown a good migration of cells in pores inside biomaterial for both biomaterials (Figure 7). Higher bone formation areas were obtained with Calciresorb biomaterial, suggesting that a biomaterial combining macroporosity and microporosity is able to promote a better osteoformation. This result is in agreement with literature showing that microporosity and macroporosity are crucial for osteoconductivity [54,55]. Moreover, the combination of HA and TCP seems to be a useful association for generating a scaffold that allows a rapid vascularization and integration during the early time point after implantation and a slow degradation [56,57]. In our study, different results in terms of proliferation, osteogenic differentiation, and osteoformation were obtained with the two kinds of biomaterials tested. In fact, proliferation assays showed that Calciresorb bone like with 100% TCP favored proliferation. However, in a biomaterial with poor proliferative potential (BM_A_), PGCAP addition seemed to improve cell proliferation (Figure 4) and allowed levels similar to those obtained with Calciresorb bone like with 100% TCP to be reached. Even if BM_F _exhibited high proliferative potential, no difference in osteogenic marker expressions was obtained between the two biomaterials tested.

The importance of enhancing the osteogenic potential of MSCs/biomaterials has been investigated by pre-culturing the seeded cells on scaffolds in the presence of osteogenic media. Results from various studies suggest that differentiation of the cells toward osteoblast lineage and secretion of bone-like extracellular matrix may have osteoinductive properties and promote *in vivo *bone formation [58-60]. In our study, alkaline phosphatase expression was upregulated in osteogenic pre-induction conditions *in vitro*. However, the expression of other osteogenic markers was not affected (Runx2) or was slightly downregulated (osteopontin, osteonectin, osteocalcin, and collagen type 1) by this pre-induction step. Results obtained *in vitro *are correlated to *in vivo *results in which little bone formation was obtained with pre-osteogenic induction. Compared with other studies, different results have been obtained and this is certainly due to osteogenic medium composition or to the difference in the length of induction period necessary to improve bone-inductive properties of constructs [32,61]. This observation raised the question of whether MSCs need to be fully differentiated in osteoblasts and which role they play in bone regeneration. Tortelli and colleagues [62] showed that the endochondral ossification process is responsible for bone formation from host origin with MSC implants but that an intramembranous ossification results from implants seeded by osteoblasts (isolated from calvaria). This group also showed that vascularization was increased in MSC implants and that bone formation was more important in osteoblast implants. Therefore, the quality and the type of ossification appear to be dependent on the origin of cells seeded on biomaterials. Other studies have confirmed this result by showing that MSCs seeded on biomaterial are able to recruit, first, host endothelial progenitors and, next, osteoprogenitor cells [63]. MSC paracrine potential might explain these results [31]. In fact, SDF-1 and its receptor CXCR4 are expressed by bone marrow MSCs and promote both proliferation and survival of these cells [64]. Furthermore, transduced MSCs secreting high levels of SDF-1 displayed an enhanced ability to form *in vivo *ectopic bone. Another study indicates that SDF-1 secreted by MSCs induced recruitment of host progenitors and promoted survival and osteogenic capacity of MSCs [65]. Adipose-derived stromal cells have also been shown to induce osteogenic differentiation of calvarial osteoblasts and to stimulate skeletal repair via paracrine signaling [66]. Moreover, MSCs loaded on subcutaneously implanted β-TCP biomaterials are able to secrete a range of cytokines in the initial post-implantation phase, which correlates with an enhancement of bone formation in a later phase of implantation [67]. This observation suggests that appropriate priming of MSCs is important to induce their secretion of specific factors at the injury site.

As Kasten and colleagues [68] showed, the addition of PRP increased alkaline phosphatase activity of undifferentiated MSC composites but had little effect on ectopic bone formation and only after 8 weeks. In agreement with our datas, their results showed that a pre-induction with chemical osteogenic inducers is not necessary for the promoting effect of PRP on osteogenesis. Moreover, it had been shown that osteogenic marker expressions were induced in MSCs expanded in PL without the need of osteogenic chemical inducers. *In vivo*, MSCs expanded in PL enhanced bone formation compared with MSCs expanded in FCS [30]. Our results also demonstrate that PGCAP by itself is able to induce osteogenic markers (type I collagen, osteonectin, osteopontin, and Runx2) and that a combination with chemical osteogenic inducers is not favorable since it decreases expression of osteogenic markers (type I collagen, osteocalcin, osteonectin, and Runx2). Reduced expression of alkaline phosphatase was also obtained in PGCAP constructs placed in osteogenic medium, as similarly shown by Bruder and colleagues [47], who demonstrate that this alteration of alkaline phosphatase expression was dependent on the PDGF/ERK pathway. *In vivo*, we have shown again that PGCAP highly enhanced bone-like tissue formation and that the addition of osteogenic medium decreased the effect of PGCAP. In conditions with PGCAP, our result showed that cells were well distributed both in glue and in pores, inside the biomaterial before implantation. This indicates that PGCAP did not seem to hinder cell migration. In conditions with PGCAP, we consider that diffusion of nutrients *in vivo *was equivalent in conditions with a pre-osteogenic induction or not. Even if cells embedded in PGCAP interacted with an environment different than those present in pores inside biomaterial, the addition of PGCAP improved bone-like tissue formations in both biomaterials (Figure 8). Future experiments should be done to test the combination of PGCAP with biomaterial and MSCs in a model of large bone defect. With a subcutaneous ectopic model of bone formation, we did not explore the impact of mechanical constraints that occur in bone defect and the impact on biomaterial degradation and further bone remodeling. However, our combination should have some interest in large bone defect. Indeed, gel formation obtained from PGCAP represents an advantage in surgical procedure to treat bone defect. PGCAP also contains various chemokines, such as SDF-1, that could play a role in host progenitor cell recruitment, as demonstrated in our study by the presence of a high number of host cells on the implanted biomaterials (data not shown). Finally, PGCAP is enriched in survival growth factors, such as HGF, IGF-1, and SDF-1, that could play an important role for improving the local survival of implanted MSCs. Furthermore, we demonstrate that PGCAP acts on proliferation and differentiation of MSCs that could be of interest in large bone defect.

## Conclusions

Our data on osteoblastic gene expression and on *in vivo *ectopic bone formation confirm that MSCs embedded in PGCAP did not require osteogenic pre-induction for exhibiting a significant osteoblastic differentiation and *in vivo *bone formation. Moreover, Calciresorb biomaterial (65% HA/35% TCP) was more powerful in promoting osteoformation. Taken together, our results indicate that the combination of Calciresorb biomaterial and PGCAP to MSCs is more efficient than the combination of Calciresorb biomaterial to osteogenic-inducted MSCs and offers an attractive alternative to autologous bone graft for bone tissue engineering.

## Abbreviations

β-TCP: beta-tricalcium phosphate; Cryo: cryoprecipitate; DAPI: 4'-6-diamidino-2-phenylindole; dsDNA: double-stranded DNA; EGF: epidermal growth factor; FCS: fetal calf serum; FGF-b: fibroblast growth factor-beta; GFP: green fluorescent protein; HA: hydroxyapatite; HGF: hepatocyte growth factor; IGF-1: insulin-like growth factor-1; IL: interleukin; MSC: mesenchymal stromal cell; i-MSC: not osteogenic induced-mesenchymal stromal cell; ni-MSC: not induced-mesenchymal stromal cell; PDGF-AB: platelet-derived growth factor-AB; PFA: paraformaldehyde; PGCAP: platelet glue obtained from cryoprecipitation of apheresis platelet products; PL: platelet lysate; PPP: platelet-poor plasma; PRF: platelet-rich fibrin; PRP: platelet-rich plasma; SDF-1a: stromal cell-derived factor 1 alpha; SEM: standard error of the mean; TC: Tissucol; TGF-b: transforming growth factor-beta; VEGF: vascular endothelial growth factor.

## Competing interests

The authors declare that they have no competing interests.

## Authors' contributions

MT participated in the study design and experimental studies and drafted the manuscript. MP participated in the management of the studies. CD participated in the conception of the study and the platelet glue analysis. IE participated in experimental studies with the platelet glue. CL-B participated in the imaging exploration analysis. PSB participated in the histological analysis. XH participated in the osteoblastic differentiation analysis. J-JL coordinated the study, revised the manuscript, and gave final approval of the version to be published. All authors read and approved the final manuscript.

## References

[B1] Cancedda R Giannoni P Mastrogiacomo M A tissue engineering approach to bone repair in large animal models and in clinical practice Biomaterials 2007 28 4240 4250 10.1016/j.biomaterials.2007.06.023 17644173

[B2] Heiple KG Goldberg VM Powell AE Bos GD Zika JM Biology of cancellous bone grafts Orthop Clin North Am 1987 18 179 185 3550570

[B3] Drosse I Volkmer E Capanna R De Biase P Mutschler W Schieker M Tissue engineering for bone defect healing: an update on a multi-component approach Injury 2008 39 Suppl 2 S9 20 18804579 10.1016/S0020-1383(08)70011-1

[B4] Dawson E Mapili G Erickson K Taqvi S Roy K Biomaterials for stem cell differentiation Adv Drug Deliv Rev 2008 60 215 228 10.1016/j.addr.2007.08.037 17997187

[B5] Chai YC Carlier A Bolander J Roberts SJ Geris L Schrooten J Van Oosterwyck H Luyten FP Current views on calcium phosphate osteogenicity and the translation into effective bone regeneration strategies Acta Biomater 2012 8 3876 3887 10.1016/j.actbio.2012.07.002 22796326

[B6] Horwitz EM Le Blanc K Dominici M Mueller I Slaper-Cortenbach I Marini FC Deans RJ Krause DS Keating A Clarification of the nomenclature for MSC: The International Society for Cellular Therapy position statement Cytotherapy 2005 7 393 395 10.1080/14653240500319234 16236628

[B7] Friedenstein AJ Gorskaja JF Kulagina NN Fibroblast precursors in normal and irradiated mouse hematopoietic organs Exp Hematol 1976 4 267 274 976387

[B8] Pittenger MF Mackay AM Beck SC Jaiswal RK Douglas R Mosca JD Moorman MA Simonetti DW Craig S Marshak DR Multilineage potential of adult human mesenchymal stem cells Science 1999 284 143 147 10.1126/science.284.5411.143 10102814

[B9] Uccelli A Moretta L Pistoia V Immunoregulatory function of mesenchymal stem cells Eur J Immunol 2006 36 2566 2573 10.1002/eji.200636416 17013987

[B10] Bielby R Jones E McGonagle D The role of mesenchymal stem cells in maintenance and repair of bone Injury 2007 38 Suppl 1 S26 32 17383482 10.1016/j.injury.2007.02.007

[B11] Kon E Muraglia A Corsi A Bianco P Marcacci M Martin I Boyde A Ruspantini I Chistolini P Rocca M Giardino R Cancedda R Quarto R Autologous bone marrow stromal cells loaded onto porous hydroxyapatite ceramic accelerate bone repair in critical-size defects of sheep long bones J Biomed Mater Res 2000 49 328 337 10.1002/(SICI)1097-4636(20000305)49:3<328::AID-JBM5>3.0.CO;2-Q 10602065

[B12] Viateau V Guillemin G Bousson V Oudina K Hannouche D Sedel L Logeart-Avramoglou D Petite H Long-bone critical-size defects treated with tissue-engineered grafts: a study on sheep J Orthop Res 2007 25 741 749 10.1002/jor.20352 17318898

[B13] Horwitz EM Prockop DJ Fitzpatrick LA Koo WW Gordon PL Neel M Sussman M Orchard P Marx JC Pyeritz RE Brenner MK Transplantability and therapeutic effects of bone marrow-derived mesenchymal cells in children with osteogenesis imperfecta Nat Med 1999 5 309 313 10.1038/6529 10086387

[B14] Le Blanc K Gotherstrom C Ringden O Hassan M McMahon R Horwitz E Anneren G Axelsson O Nunn J Ewald U Nordén-Lindeberg S Jansson M Dalton A Aström E Westgren M Fetal mesenchymal stem-cell engraftment in bone after in utero transplantation in a patient with severe osteogenesis imperfecta Transplantation 2005 79 1607 1614 10.1097/01.TP.0000159029.48678.93 15940052

[B15] Quarto R Mastrogiacomo M Cancedda R Kutepov SM Mukhachev V Lavroukov A Kon E Marcacci M Repair of large bone defects with the use of autologous bone marrow stromal cells N Engl J Med 2001 344 385 386 10.1056/NEJM200102013440516 11195802

[B16] Marcacci M Kon E Moukhachev V Lavroukov A Kutepov S Quarto R Mastrogiacomo M Cancedda R Stem cells associated with macroporous bioceramics for long bone repair: 6- to 7-year outcome of a pilot clinical study Tissue Eng 2007 13 947 955 10.1089/ten.2006.0271 17484701

[B17] Doucet C Ernou I Zhang Y Llense JR Begot L Holy X Lataillade JJ Platelet lysates promote mesenchymal stem cell expansion: a safety substitute for animal serum in cell-based therapy applications J Cell Physiol 2005 205 228 236 10.1002/jcp.20391 15887229

[B18] Anitua E Sanchez M Zalduendo MM de la Fuente M Prado R Orive G Andia I Fibroblastic response to treatment with different preparations rich in growth factors Cell Prolif 2009 42 162 170 10.1111/j.1365-2184.2009.00583.x 19250293 PMC6496288

[B19] Visser LC Arnoczky SP Caballero O Kern A Ratcliffe A Gardner KL Growth factor-rich plasma increases tendon cell proliferation and matrix synthesis on a synthetic scaffold: an in vitro study Tissue Eng Part A 2010 16 1021 1029 10.1089/ten.tea.2009.0254 19839921

[B20] Marx RE Carlson ER Eichstaedt RM Schimmele SR Strauss JE Georgeff KR Platelet-rich plasma: Growth factor enhancement for bone grafts Oral Surg Oral Med Oral Pathol Oral Radiol Endod 1998 85 638 646 10.1016/S1079-2104(98)90029-4 9638695

[B21] Nurden AT Nurden P Sanchez M Andia I Anitua E Platelets and wound healing Front Biosci 2008 13 3532 3548 18508453 10.2741/2947

[B22] Intini G The use of platelet-rich plasma in bone reconstruction therapy Biomaterials 2009 30 4956 4966 10.1016/j.biomaterials.2009.05.055 19573909

[B23] Rai B Oest ME Dupont KM Ho KH Teoh SH Guldberg RE Combination of platelet-rich plasma with polycaprolactone-tricalcium phosphate scaffolds for segmental bone defect repair J Biomed Mater Res A 2007 81 888 899 17236215 10.1002/jbm.a.31142

[B24] Ranly DM Lohmann CH Andreacchio D Boyan BD Schwartz Z Platelet-rich plasma inhibits demineralized bone matrix-induced bone formation in nude mice J Bone Joint Surg Am 2007 89 139 147 10.2106/JBJS.F.00388 17200321

[B25] Giovanini AF Deliberador TM Gonzaga CC de Oliveira Filho MA Gohringer I Kuczera J Zielak JC de Andrade Urban C Platelet-rich plasma diminishes calvarial bone repair associated with alterations in collagen matrix composition and elevated CD34+ cell prevalence Bone 2010 46 1597 1603 10.1016/j.bone.2010.02.026 20206725

[B26] Hakimi M Jungbluth P Sager M Betsch M Herten M Becker J Windolf J Wild M Combined use of platelet-rich plasma and autologous bone grafts in the treatment of long bone defects in mini-pigs Injury 2010 41 811 817 10.1016/j.injury.2009.12.005 20097341

[B27] Kitoh H Kitakoji T Tsuchiya H Katoh M Ishiguro N Transplantation of culture expanded bone marrow cells and platelet rich plasma in distraction osteogenesis of the long bones Bone 2007 40 522 528 10.1016/j.bone.2006.09.019 17070744

[B28] Pieri F Lucarelli E Corinaldesi G Iezzi G Piattelli A Giardino R Bassi M Donati D Marchetti C Mesenchymal stem cells and platelet-rich plasma enhance bone formation in sinus grafting: a histomorphometric study in minipigs J Clin Periodontol 2008 35 539 546 10.1111/j.1600-051X.2008.01220.x 18422697

[B29] Chai YC Roberts SJ Desmet E Kerckhofs G van Gastel N Geris L Carmeliet G Schrooten J Luyten FP Mechanisms of ectopic bone formation by human osteoprogenitor cells on CaP biomaterial carriers Biomaterials 2012 33 3127 3142 10.1016/j.biomaterials.2012.01.015 22269651

[B30] Chevallier N Anagnostou F Zilber S Bodivit G Maurin S Barrault A Bierling P Hernigou P Layrolle P Rouard H Osteoblastic differentiation of human mesenchymal stem cells with platelet lysate Biomaterials 2010 31 270 278 10.1016/j.biomaterials.2009.09.043 19783038

[B31] Tasso R Gaetani M Molino E Cattaneo A Monticone M Bachi A Cancedda R The role of bFGF on the ability of MSC to activate endogenous regenerative mechanisms in an ectopic bone formation model Biomaterials 2012 33 2086 2096 10.1016/j.biomaterials.2011.11.043 22169138

[B32] Castano-Izquierdo H Alvarez-Barreto J van den Dolder J Jansen JA Mikos AG Sikavitsas VI Pre-culture period of mesenchymal stem cells in osteogenic media influences their in vivo bone forming potential J Biomed Mater Res A 2007 82 129 138 17269144 10.1002/jbm.a.31082

[B33] Scott MA Levi B Askarinam A Nguyen A Rackohn T Ting K Soo C James AW Brief review of models of ectopic bone formation Stem Cells Dev 2012 21 655 667 10.1089/scd.2011.0517 22085228 PMC3295855

[B34] Llames SG Del Rio M Larcher F Garcia E Garcia M Escamez MJ Jorcano JL Holguin P Meana A Human plasma as a dermal scaffold for the generation of a completely autologous bioengineered skin Transplantation 2004 77 350 355 10.1097/01.TP.0000112381.80964.85 14966407

[B35] Vandesompele J De Preter K Pattyn F Poppe B Van Roy N De Paepe A Speleman F Accurate normalization of real-time quantitative RT-PCR data by geometric averaging of multiple internal control genes Genome Biol 2002 3 RESEARCH0034 10.1186/gb-2002-3-7-research0034 PMC126239 12184808

[B36] Winter A Breit S Parsch D Benz K Steck E Hauner H Weber RM Ewerbeck V Richter W Cartilage-like gene expression in differentiated human stem cell spheroids: a comparison of bone marrow-derived and adipose tissue-derived stromal cells Arthritis Rheum 2003 48 418 429 10.1002/art.10767 12571852

[B37] Anitua E The use of plasma-rich growth factors (PRGF) in oral surgery Pract Proced Aesthet Dent 2001 13 487 493 quiz 487-493 11544821

[B38] Dugrillon A Eichler H Kern S Kluter H Autologous concentrated platelet-rich plasma (cPRP) for local application in bone regeneration Int J Oral Maxillofac Surg 2002 31 615 619 10.1054/ijom.2002.0322 12521317

[B39] Thorn JJ Sorensen H Weis-Fogh U Andersen M Autologous fibrin glue with growth factors in reconstructive maxillofacial surgery Int J Oral Maxillofac Surg 2004 33 95 100 10.1054/ijom.2003.0461 14690664

[B40] Dohan DM Choukroun J Diss A Dohan SL Dohan AJ Mouhyi J Gogly B Platelet-rich fibrin (PRF): a second-generation platelet concentrate. Part I: technological concepts and evolution Oral Surg Oral Med Oral Pathol Oral Radiol Endod 2006 101 e37 44 10.1016/j.tripleo.2005.07.008 16504849

[B41] Callum JL Karkouti K Lin Y Cryoprecipitate: the current state of knowledge Transfus Med Rev 2009 23 177 188 10.1016/j.tmrv.2009.03.001 19539873

[B42] Vogler EA Siedlecki CA Contact activation of blood-plasma coagulation Biomaterials 2009 30 1857 1869 10.1016/j.biomaterials.2008.12.041 19168215 PMC2705825

[B43] Bieback K Hecker A Kocaomer A Lannert H Schallmoser K Strunk D Kluter H Human alternatives to fetal bovine serum for the expansion of mesenchymal stromal cells from bone marrow Stem Cells 2009 27 2331 2341 10.1002/stem.139 19544413

[B44] Weibrich G Hansen T Kleis W Buch R Hitzler WE Effect of platelet concentration in platelet-rich plasma on peri-implant bone regeneration Bone 2004 34 665 671 10.1016/j.bone.2003.12.010 15050897

[B45] Gawaz M Langer H May AE Platelets in inflammation and atherogenesis J Clin Invest 2005 115 3378 3384 10.1172/JCI27196 16322783 PMC1297269

[B46] Kratchmarova I Blagoev B Haack-Sorensen M Kassem M Mann M Mechanism of divergent growth factor effects in mesenchymal stem cell differentiation Science 2005 308 1472 1477 10.1126/science.1107627 15933201

[B47] Gruber R Karreth F Kandler B Fuerst G Rot A Fischer MB Watzek G Platelet-released supernatants increase migration and proliferation, and decrease osteogenic differentiation of bone marrow-derived mesenchymal progenitor cells under in vitro conditions Platelets 2004 15 29 35 10.1080/09537100310001643999 14985174

[B48] Ng F Boucher S Koh S Sastry KS Chase L Lakshmipathy U Choong C Yang Z Vemuri MC Rao MS Tanavde V PDGF, TGF-beta, and FGF signaling is important for differentiation and growth of mesenchymal stem cells (MSCs): transcriptional profiling can identify markers and signaling pathways important in differentiation of MSCs into adipogenic, chondrogenic, and osteogenic lineages Blood 2008 112 295 307 10.1182/blood-2007-07-103697 18332228

[B49] Bao P Kodra A Tomic-Canic M Golinko MS Ehrlich HP Brem H The role of vascular endothelial growth factor in wound healing J Surg Res 2009 153 347 358 10.1016/j.jss.2008.04.023 19027922 PMC2728016

[B50] Keramaris NC Calori GM Nikolaou VS Schemitsch EH Giannoudis PV Fracture vascularity and bone healing: a systematic review of the role of VEGF Injury 2008 39 Suppl 2 S45 57 18804573 10.1016/S0020-1383(08)70015-9

[B51] Massberg S Konrad I Schurzinger K Lorenz M Schneider S Zohlnhoefer D Hoppe K Schiemann M Kennerknecht E Sauer S Schulz C Kerstan S Rudelius M Seidl S Sorge F Langer H Peluso M Goyal P Vestweber D Emambokus NR Busch DH Frampton J Gawaz M Platelets secrete stromal cell-derived factor 1alpha and recruit bone marrow-derived progenitor cells to arterial thrombi in vivo J Exp Med 2006 203 1221 1233 10.1084/jem.20051772 16618794 PMC2121205

[B52] Malard O Bouler JM Guicheux J Heymann D Pilet P Coquard C Daculsi G Influence of biphasic calcium phosphate granulometry on bone ingrowth, ceramic resorption, and inflammatory reactions: preliminary in vitro and in vivo study J Biomed Mater Res 1999 46 103 111 10.1002/(SICI)1097-4636(199907)46:1<103::AID-JBM12>3.0.CO;2-Z 10357141

[B53] Luong-Van E Grondahl L Song S Nurcombe V Cool S The in vivo assessment of a novel scaffold containing heparan sulfate for tissue engineering with human mesenchymal stem cells J Mol Histol 2007 38 459 468 10.1007/s10735-007-9129-y 17694276

[B54] Habibovic P Yuan H van der Valk CM Meijer G van Blitterswijk CA de Groot K 3D microenvironment as essential element for osteoinduction by biomaterials Biomaterials 2005 26 3565 3575 10.1016/j.biomaterials.2004.09.056 15621247

[B55] Yuan H Fernandes H Habibovic P de Boer J Barradas AM de Ruiter A Walsh WR van Blitterswijk CA de Bruijn JD Osteoinductive ceramics as a synthetic alternative to autologous bone grafting Proc Natl Acad Sci USA 2010 107 13614 13619 10.1073/pnas.1003600107 20643969 PMC2922269

[B56] Ghanaati S Barbeck M Orth C Willershausen I Thimm BW Hoffmann C Rasic A Sader RA Unger RE Peters F Kirkpatrick CJ Influence of beta-tricalcium phosphate granule size and morphology on tissue reaction in vivo Acta Biomater 2010 6 4476 4487 10.1016/j.actbio.2010.07.006 20624495

[B57] Ghanaati S Barbeck M Detsch R Deisinger U Hilbig U Rausch V Sader R Unger RE Ziegler G Kirkpatrick CJ The chemical composition of synthetic bone substitutes influences tissue reactions in vivo: histological and histomorphometrical analysis of the cellular inflammatory response to hydroxyapatite, beta-tricalcium phosphate and biphasic calcium phosphate ceramics Biomed Mater 2012 7 015005 10.1088/1748-6041/7/1/015005 22287541

[B58] Kirker-Head C Karageorgiou V Hofmann S Fajardo R Betz O Merkle HP Hilbe M von Rechenberg B McCool J Abrahamsen L Nazarian A Cory E Curtis M Kaplan D Meinel L BMP-silk composite matrices heal critically sized femoral defects Bone 2007 41 247 255 10.1016/j.bone.2007.04.186 17553763 PMC2695963

[B59] Li H Dai K Tang T Zhang X Yan M Lou J Bone regeneration by implantation of adipose-derived stromal cells expressing BMP-2 Biochem Biophys Res Commun 2007 356 836 842 10.1016/j.bbrc.2007.02.165 17391646

[B60] Yoon E Dhar S Chun DE Gharibjanian NA Evans GR In vivo osteogenic potential of human adipose-derived stem cells/poly lactide-co-glycolic acid constructs for bone regeneration in a rat critical-sized calvarial defect model Tissue Eng 2007 13 619 627 10.1089/ten.2006.0102 17518608

[B61] Song IH Caplan AI Dennis JE In vitro dexamethasone pretreatment enhances bone formation of human mesenchymal stem cells in vivo J Orthop Res 2009 27 916 921 10.1002/jor.20838 19137580

[B62] Tortelli F Tasso R Loiacono F Cancedda R The development of tissue-engineered bone of different origin through endochondral and intramembranous ossification following the implantation of mesenchymal stem cells and osteoblasts in a murine model Biomaterials 2010 31 242 249 10.1016/j.biomaterials.2009.09.038 19796807

[B63] Tasso R Fais F Reverberi D Tortelli F Cancedda R The recruitment of two consecutive and different waves of host stem/progenitor cells during the development of tissue-engineered bone in a murine model Biomaterials 2010 31 2121 2129 10.1016/j.biomaterials.2009.11.064 20004968

[B64] Kortesidis A Zannettino A Isenmann S Shi S Lapidot T Gronthos S Stromal-derived factor-1 promotes the growth, survival, and development of human bone marrow stromal stem cells Blood 2005 105 3793 3801 10.1182/blood-2004-11-4349 15677562

[B65] Otsuru S Tamai K Yamazaki T Yoshikawa H Kaneda Y Circulating bone marrow-derived osteoblast progenitor cells are recruited to the bone-forming site by the CXCR4/stromal cell-derived factor-1 pathway Stem Cells 2008 26 223 234 10.1634/stemcells.2007-0515 17932420

[B66] Levi B James AW Nelson ER Li S Peng M Commons GW Lee M Wu B Longaker MT Human adipose-derived stromal cells stimulate autogenous skeletal repair via paracrine hedgehog signaling with calvarial osteoblasts Stem Cells Dev 2011 20 243 257 10.1089/scd.2010.0250 20698749 PMC3128781

[B67] Byeon YE Ryu HH Park SS Koyama Y Kikuchi M Kim WH Kang KS Kweon OK Paracrine effect of canine allogenic umbilical cord blood-derived mesenchymal stromal cells mixed with beta-tricalcium phosphate on bone regeneration in ectopic implantations Cytotherapy 2010 12 626 636 10.3109/14653249.2010.481665 20438297

[B68] Kasten P Vogel J Luginbuhl R Niemeyer P Weiss S Schneider S Kramer M Leo A Richter W Influence of platelet-rich plasma on osteogenic differentiation of mesenchymal stem cells and ectopic bone formation in calcium phosphate ceramics Cells Tissues Organs 2006 183 68 79 10.1159/000095511 17053323

